# Linguistic, visuospatial, and kinematic writing characteristics in cognitively impaired patients with beta-amyloid deposition

**DOI:** 10.3389/fnagi.2023.1217746

**Published:** 2023-09-11

**Authors:** Seo Kyung An, Hyemin Jang, Hee Jin Kim, Duk L. Na, Ji Hye Yoon

**Affiliations:** ^1^Department of Speech-Language Pathology and Audiology, Hallym University, Chuncheon, Republic of Korea; ^2^Department of Neurology, Samsung Medical Center, Sungkyunkwan University School of Medicine, Seoul, Republic of Korea; ^3^Division of Speech Pathology and Audiology, Research Institute of Audiology and Speech Pathology, Hallym University, Chuncheon, Republic of Korea

**Keywords:** beta amyloid, cognitive impairment, word dictation, writing, speed

## Abstract

**Introduction:**

Beta-amyloid (Aβ) deposition, a hallmark of Alzheimer’s disease (AD), begins before dementia and is an important factor in mild cognitive impairment (MCI). Aβ deposition is a recognized risk factor for various cognitive impairments and has been reported to affect motor performance as well. This study aimed to identify the linguistic, visuospatial, and kinematic characteristics evident in the writing performance of patients with cognitive impairment (CI) who exhibit Aβ deposition.

**Methods:**

A total of 31 patients diagnosed with amnestic mild cognitive impairment (aMCI) with Aβ deposition, 26 patients with Alzheimer’s-type dementia, and 33 healthy control (HC) participants without deposition were administered tasks involving dictation of 60 regular words, irregular words, and non-words consisting of 1–4 syllables. Responses from all participants were collected and analyzed through digitized writing tests and analysis tools.

**Results:**

In terms of linguistic aspects, as cognitive decline progressed, performance in the dictation of irregular words decreased, with errors observed in substituting the target grapheme with other graphemes. The aMCI group frequently exhibited corrective aspects involving letter rewriting during the task. In terms of visuospatial aspects, the AD group displayed more errors in grapheme combination compared to the HC group. Lastly, in the kinematic aspects, both the aMCI group and the AD group exhibited slower writing speeds compared to the HC group.

**Discussion:**

The findings suggest that individuals in the CI group exhibited lower performance in word dictation tasks than those in the HC group, and these results possibly indicate complex cognitive-language-motor deficits resulting from temporal-parietal lobe damage, particularly affecting spelling processing. These results provide valuable clinical insights into understanding linguistic-visuospatial-kinematic aspects that contribute to the early diagnosis of CI with Aβ deposition.

## 1. Introduction

Beta amyloid (Aβ) deposition is recognized as a key pathological feature of Alzheimer’s disease (AD), a degenerative disease. Aβ deposition can result in axonal injury, synaptic dysfunction, and reduction of neuronal connections and acetylcholine (Richards and Sabbagh, 2014; Walhovd et al., 2014). Aβ is deposited as plaques starting in the basal temporal and orbitofrontal neocortex, followed by deposition in the allocortex and amygdala. Next, plaques are deposited in almost all higher association areas of the neocortex, expanding further into secondary neocortical areas and into the striatum, until they are in virtually all areas of the neocortex and the mesencephalon, eventually reaching the lower brainstem and cerebellar cortex (Thal et al., 2002; Braak and Del Tredici, 2015). With the advancement of molecular imaging technology, Aβ deposition in the brain can now be visualized and confirmed through positron emission tomography (PET), thereby enabling the discovery of pathological factors that may predict cognitive decline (Lim et al., 2013; Donohue et al., 2014; Kang and Lim, 2018). Aβ deposition has been observed to begin approximately 15–20 years before the onset of AD (Jeon et al., 2016; Kang and Lim, 2018), and may also occur in amnestic mild cognitive impairment (aMCI), a precursor to AD (Wolk and Klunk, 2009; Kang and Lim, 2018). It has been reported that patients diagnosed with aMCI who exhibit positive (+) Aβ deposition have lower cognitive abilities and a higher risk of conversion to AD compared to those with negative (−) findings (Lim et al., 2013; Donohue et al., 2014). Therefore, it is important to identify the characteristics of cognitive impairment (CI) in patients who display Aβ deposition along a continuous spectrum of regression through various novel tasks or methods (Petersen et al., 2001). The initial hallmark symptom of AD is typically episodic memory impairment, affecting cognitive functions related to memory (Salmon and Bondi, 2009). As the deposition progresses, deficits in name retrieval, calculation ability, and disorientation may manifest (Jeong and Na, 2003; Jeon et al., 2016). Additionally, recall of motor representations for initiating movements and generating appropriate motor commands can be impaired, leading to defects in motor function (Mollica et al., 2019).

The decline in cognitive and motor functions can significantly impact language abilities, including spelling processes and writing activities, which consist of interactions between attention, memory, visuospatial ability, and graphomotor control ability (Singer and Bashir, 2004; Mangen and Velay, 2010). In terms of language, patients with early-onset AD often exhibit difficulties in writing irregular words, words in which phoneme and grapheme do not confer, which may be attributed to damage in the lexical route (Appell et al., 1982; Cummings et al., 1986; Aarsland et al., 1996; Sung, 2010); patients also encounter challenges in writing non-words (Platel et al., 1993; Silveri et al., 2007). In terms of visuospatial aspects, omission or addition of strokes in letters were observed among patients with AD (Yoon et al., 2012; Yu and Kim, 2014). In the motor aspects, patients with AD were observed to have unrecognizable writing patterns due to a decreased graphomotor control ability (Harnish and Neils-Strunjas, 2008; Delazer et al., 2021). Furthermore, since writing requires holding a writing instrument, hand grip strength, which corresponds to the force used to hold or grip an object, may impact writing performance. Accordingly, measurements of grip strength in CI groups, including patients with MCI and AD, have confirmed weaker grip strength compared to those in the healthy control group (Ishihara et al., 2019; Su et al., 2021; Jockusch et al., 2022). In total, this suggests that cognitive functions such as language ability, visuospatial perception, and working memory are correlated with grip strength, and that grip strength may serve as a predictor of cognitive function in patients with MCI and AD (Buchman et al., 2007; Boyle et al., 2010; Sternäng et al., 2016; Su et al., 2021).

Writing tasks, which consist of diverse functions, can be a good medium to simultaneously identify the aspects of decline in cognitive and motor abilities in patients with AD and aMCI with Aβ deposition. However, previous studies (Appell et al., 1982; Cummings et al., 1986; Platel et al., 1993; Aarsland et al., 1996; Silveri et al., 2007; Harnish and Neils-Strunjas, 2008; Sung, 2010; Yoon et al., 2012; Yu and Kim, 2014; Delazer et al., 2021) investigating the linguistic, visuospatial, and motor writing characteristics of patients with AD have only targeted those diagnosed through cognitive function tests without verifying the presence of Aβ deposition. Notably, clinical manifestations may vary depending on the presence or absence of Aβ deposition (Chételat et al., 2016; Jeon et al., 2016; Tomadesso et al., 2018), and these manifestations may reflect onto writing ability; thus, confirming the presence or absence of Aβ deposition is necessary for clinical application and generalization of results.

With the advancement of objective measuring and analyzing systems for movement, previous studies have assessed the kinematic aspects of handwriting. In the case of writing time, written latencies and duration of writing movement were measured (Müller et al., 2017; Afonso et al., 2019), or the speed was measured considering the length of the stroke (Werner et al., 2006; Yan et al., 2008). However, in some previous studies using tablet computers and digital pens based on electromagnetic resonance technology, participants were unable to directly write letters while looking at the tablet screen, but rather, wrote letters on a separate tablet while viewing a connected monitor. Therefore, the ability to integrate vision and movement possibly influenced performance.

More recently, there have been attempts to confirm the characteristics observed in the handwriting of cognitively disabled individuals by using various dynamic information, such as pen pressure and time/speed aspects. Researchers extracted both dynamic and static features, such as the velocity and total length of strokes, from the data (Cilia et al., 2021a). These features were measured in on-paper (features extracted from written traits) and in-air (planning activity for positioning the pen tip when the pen is lifted from the sheet) categories during graphic drawing, copying, memorizing words, and dictation tasks (e.g., simple sentences and telephone numbers). Additionally, researchers tested whether combining shape and dynamic features using an online handwriting database enables the diagnosis of AD (Cilia et al., 2021b). They generated an offline synthetic color image for each feature and demonstrated that kinematic handwriting analysis can be helpful in supporting the early diagnosis of CI. Furthermore, to address limitations due to a small number of participants performing a few tasks, the study suggested a standardized experimental protocol for data collection and provided handwriting samples from many people affected by AD (Cilia et al., 2022). The measured parameters included (x, y) coordinates, pressure, azimuth angle, altitude, displacement, velocity, and acceleration.

Since previous studies were more focused on assessing kinematic aspects rather than cognitive-linguistic aspects, lots of geometrical drawings and tasks involving the copying of words or numbers were performed. Assessments for geometrical drawings were made to confirm the automatization and coordination of wrist joint, finger joint, and hand movements through tasks such as connecting horizontal and vertical lines or drawing circles. For copying tasks, subcategories of word conditions such as regular and non-words (Cilia et al., 2020) or regular, irregular, and non-words (Cilia et al., 2022) were used. However, the characteristics of various routes for spelling and writing may be better investigated through a writing-to-dictation task rather than a copying task. Therefore, although objective measurement and analysis methods for kinematic aspects based on the previous studies will be utilized, our primary objective is to identify which pathways are difficult to use in the ‘writing-to-dictation task’ of Korean CI patients with Aβ deposition.

**FIGURE 1:**
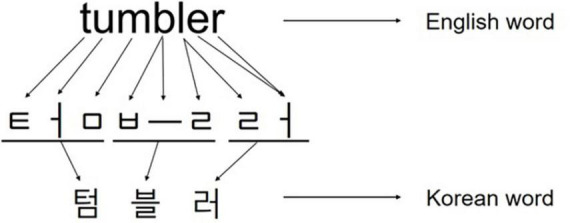
Arrangement of graphemes in English words and graphemes in Korean words.

Furthermore, the previous studies were predominantly conducted in Western languages that use the alphabet, making it challenging to generalize the results to Korean participants who use the Korean writing system (Hangeul). Hangeul, like the English alphabet, is a phonographic language (phonogram) in which one sound is represented by one grapheme, but it differs from the alphabet system in terms of visuospatial aspects (Yoon et al., 2013; Park, 2016). Unlike the alphabet system where consonants and vowels are arranged horizontally, the Hangeul system follows a written language system with distinct visuospatial characteristics in which consonants and vowels are spatially arranged within a syllable. (a) vertical- (arranged from top-to-bottom, e.g. 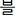), (b) horizontal- (arranged from left-to-right, e.g. 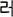), and (c) mixed-construction (i.e., combination of horizontal and vertical constructions, e.g. 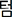). Each syllable in Hangeul is visually separated (Yoon et al., 2013; Figure 1). Therefore, assessing changes in performance according to the length of Hangeul syllables is essential, as the language-specific syllable structure of Hangeul may affect the visuospatial and kinematic elements as well as linguistic elements of writing.

Therefore, this study aimed to investigate the impact of Aβ deposition, a pathological factor, in confirming the characteristics of linguistic, visuospatial, and motor abilities among individuals with CI using a word dictation task. To investigate the linguistic aspects of writing routes, the dictation task consisted of regular words, irregular words, and non-words. To examine the visuospatial aspects of Hangeul writing, diverse combinations of graphemes were included in the dictation task. With respect to the motor (kinematic) elements of writing, pen pressure and writing speed during task performance were objectively and quantitatively measured using a digitized software program. Moreover, hand grip strength, which is an index of basic hand power that can affect writing, was measured by hand grip. Lastly, based on the uniqueness of Hangeul, this study attempted to examine kinematic elements (pen pressure, writing speed) according to the length of syllables in word dictation.

## 2. Materials and methods

### 2.1. Participants

A total of 90 adults aged ≥50 years old participated in this study. All CI participants were diagnosed with a degenerative disease, of whom 26 were diagnosed with AD (male:female = 10:16) and 31 were diagnosed with aMCI (male:female = 8:23), based on neurological examination and medical history at the general hospital. Additionally, 33 adults with normal cognitive function (HC) (male:female = 9:24) were included. Aβ deposition was diagnosed by visual evaluation by a nuclear medicine physician.

The selection criteria for each group are as follows:

The AD group included (1) those whose Aβ denatured protein accumulation was confirmed as positive (+) on PET, (2) those whose standard score was less than 16th percentile based on age and years of education as a result of Seoul Neuropsychological Screening Battery-2, SNSB-II (Kang et al., 2012), (3) Clinical Dementia Rating scale (CDR) of mild dementia, (4) those with no visual, hearing, or hand-motor impairments that affected task performance, (5) right-handed individuals, and (6) adults with educational attainment of primary school or higher.

The aMCI group included (1) those whose Aβ denatured protein accumulation was confirmed as positive (+) on PET, (2) those whose SNSB-II (Kang et al., 2012) result, delayed recall score of the Seoul Verbal Learning Test, or delayed recall score of the Rey Figure Test was less than 16th percentile, (3) those with familial history of cognitive impairment, (4) those with no visual, hearing, or hand-motor impairments that affected task performance, (5) right-handed individuals, and (6) adults with educational attainment of primary school or higher.

The HC group included (1) those whose Aβ denatured protein accumulation was confirmed as negative (−) on PET, (2) those whose SNSB-II (Kang et al., 2012) results, age, and years of education are equivalent to 16th percentile or higher in all areas of cognitive function, (3) those with no history of mental or neurological disease after conducting a health screening questionnaire (Christensen et al., 1991), (4) those who scored <8 points on the Short form Geriatric Depression Scale (SGDS) and had a normal level of depression, (5) those with no visual, hearing, or hand-motor impairments that affected task performance, (6) right-handed individuals, and (7) adults with educational attainment of primary school or higher.

The Korean-Mini Mental State Examination (K-MMSE) (Kang, 2006) and the Korean Version of the Montreal Cognitive Assessment (MoCA-K) (Lee et al., 2008) were used to check the overall cognitive level of the participants before the test. There were no significant differences among the three groups in terms of years of education [*F*_(2,87)_ = 1.865, *p* = 0.161], age [*F*_(2,87)_ = 0.284, *p* = 0.753] and SGDS scores [*F*_(2,87)_ = 2.415, *p* = 0.095] (Table 1). A total of 67 participants (21 in the HC group, 25 in the aMCI group, and 21 in the AD group) were tested for grip strength, which is thought to be related to writing tasks, and the results showed no difference among the three groups [*F*_(2,64)_ = 0.194, *p* = 0.824].

**TABLE 1:** Participants’ characteristics.

Characteristic	HC (*n* = 33)	aMCI (*n* = 31)	AD (*n* = 26)	*F*
Age (years)	71.24 (7.28)	72.23 (7.15)	70.73 (8.73)	0.753
Education (years)	12.42 (3.97)	14.19 (3.23)	12.73 (4.39)	1.865
K-MMSE (score)	29.09 (0.98)	24.96 (2.79)	19.69 (3.26)	103.586***
MoCA-K (score)	27.21 (2.15)	20.06 (3.76)	14.85 (4.17)	98.616***
SGDS (score)	3.06 (2.03)	2.48 (2.53)	1.85 (1.59)	2.415
Hand grip (kg)	26.66 (9.56)	26.02 (6.34)	27.71 (11.45)	0.194

HC, healthy control; aMCI, amnestic mild cognitive impairment; AD, Alzheimer’s disease; M, male; F, female; K-MMSE, Korean-Mini Mental State Examination; MoCA-K, Korean Version of the Montreal Cognitive Assessment; SGDS, Short-form Geriatric Depression Scale; values are presented as mean (SD). ***p < 0.001.

### 2.2. Research tools and research tasks

#### 2.2.1. Research tools

For the mechanical measurement of the writing task, a tablet laptop (Samsung Galaxybook Flex 2, Republic of Korea) and a stylus (Staedtler Noris, Germany) designed in the shape of a real pencil that uses electromagnetic resonance technology were used. For an objective and automatic analysis of writing, the Brief Agraphia Test software (Yoon and Na, in press a) was used which measures pen pressure, stroke length, duration, and the coordinates of each performance. Additionally, to objectively measure hand motor performance, a grip strength measuring instrument (Jamar Plus + Hand Dynamometer, Serial No. 2020120624) was used.

#### 2.2.2. Research tasks

The research task consisted of various tasks. For the dictation tasks, a total of 60 words were selected, consisting of 20 each of regular words, irregular words, and non-words with 1 to 4 syllables composed of various phonemes (Yoon and Na, in press b). For word selection, we referred to the Vocabulary Frequency Dictionary (Kim, 2005), and among a total of 82,501 vocabularies, the top 30% of high-frequency words and the bottom 30% of low-frequency words were selected. Cases corresponding to the middle were designated as medium-frequency words, and the ratio of each frequency was included equally. Regular words were defined as having a matching grapheme and phoneme and included diphthongs. Among the diphthongs, 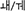 and, 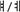 whose pronunciation was not differentiated in actual speech, were excluded. In the case of irregular words, 7 phonological changes (tensification, aspiration, palatalization, nasalization, lateralization, consonant cluster simplification, and neutralization of plosives) were included. The Hangeul 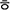 deletion and “Sai-Sori (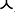)” phenomena were excluded because of their less frequent use. To examine the visuospatial aspects of writing, horizontal (transverse) writing in which grapheme elements are arranged from left to right in consideration of the visuospatial characteristics of Hangeul [e.g., 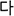 (/da/)], vertical (longitudinal) writing in which grapheme elements are arranged from top to bottom [e.g., 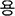 (/yong/means “dragon”)], and vertical and horizontal mixed writing [e.g., 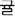 (/gyul/means “mandarin”)], to maintain an even direction in the arrangement of grapheme included for each composed word.

An additional task of copying was performed in which the participants imitated the visually presented letter as how they were perceived. This task was included because we observed that the pen pressure and writing speed of the participants were affected in various ways as the syllable length increased in the word dictation task. The word dictation tasks can be affected by cognitive and language ability because it is necessary to listen and memorize the words spoken at once due to the nature of the test. Particularly, when the length of syllables increases, the number of syllables to be memorized increases, which can cause a load on the working memory, leading to impacts on pen pressure and writing speed. Therefore, to exclude the influence of working memory in terms of kinematics, 16 of the 90 participants (9 in the HC group, 4 in the aMCI group, and 3 in the AD group) were administered the additional tasks. For the copying task, a total of 30 words were randomly selected, 10 each for each word type, among the word dictation questions. The font selected was Nanum Gothic based on previous research that confirmed the readability of Hangeul on this font (Hwang et al., 1997; Song et al., 2009). The font size used was 72 pt, and the word was presented at the top center of the tablet laptop screen. All words for the tasks (dictation and copying) are presented in Supplementary Table 1.

### 2.3. Research procedure

Data collection for this study was conducted on patients and healthy individuals who visited the neurology department of a general hospital in Seoul. Before conducting the writing test, the cognitive function evaluation (SNSB-II) results of all participants who had consented to participate in the study were checked on the electronic medical record form. The collected data included the results of the cognitive function evaluation conducted within the last year, a health screening questionnaire, and an abbreviated geriatric depression scale (SGDS) conducted as screening tests prior to the main test. To check the cognitive abilities of the participants, the Korean version of the Mini-Mental State Examination (K-MMSE) and the Korean version of the Montreal Cognitive Assessment (MoCA-K) were administered for 10 to 15 min. After the screening test, the participants were given a short break and afterward the main test was conducted. The main test was a word dictation task and was conducted in the order of regular word, irregular word, and non-words. Cognitive ability tests and dictation tasks require quiet environments with ample space because these tasks require listening to the stimuli presented in the auditory sense and answering or dictating them in spoken words. Therefore, the test was conducted in a conference room located in the hospital at a 1:1 participant-to-facilitator ratio. The succeeding test was the grip strength measurement and the additional copying task was conducted on select participants. The total examination time, including the screening test and additional tasks, took approximately 40 min. All procedures in this study were conducted after receiving prior approval (No. 2021-12-068) from the Institutional Review Board of the general hospital.

All writing tasks were performed by the participants on a tablet laptop screen while sitting on a chair with the correct posture and holding the stylus as if holding a pencil. Since the stimulus for the word dictation task can be affected by the volume or speaking speed of the examiner, only one examiner conducted the study. All words were presented with a volume of about 55–60 dB SPL, and the presentation speed was presented in spoken language by checking the average speed (about 510 ms) calculated in syllable units after the examiner called each word once through the speech analysis program Praat. Before starting the dictation task, the examiner asked the participants to listen to the words presented auditorily and speak along to check whether the words were recognized. However, since one-syllable irregular words can be misinterpreted as meaningless non-words if presented without context, meaning clues were presented together but limited to one-syllable irregular words [e.g., 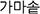 (/gamasot/means “iron pot”) so 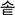 (/sot/) should be written]. In the case of non-words, low familiarity and difficulty in accessing the meaning can lead to questioning of the task performance. Additionally, auditory misperceptions may occur during the task, such as hearing and writing down the phonemes of word stimuli as similar sounds [e.g., 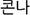 (/konna/) as 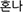 (/honna/)]. To prevent this, the examiner provided a disclaimer that stated, “I will now play some strange words that do not exist in Korean. Please repeat what you hear, and if it is correct, we will proceed with the dictation task in order.” In the case of non-words, auditory misperceptions may also occur for high-frequency consonants such as, /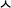/, /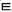/ and, /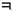/, as well as diphthongs. When meaning clues are provided [e.g., When writing 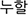 (/nuhal/) as 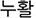 (/nuhwal/)], correct responses may be influenced by the utilization of the semantic system. To exclude this, rather, meaning clues on the syllable that showed the wrong response was provided and clarified the target syllable was not the same to the wrong syllable. For the copying task, the instruction given was, “Please copy the characters presented on the screen in the space below.”

The posture for measuring grip strength was based on previous studies (Guerra et al., 2017), in which the participants sat on a chair with their arms fixed at a 90° angle to the side of their body. The handle was set at Level II, regardless of hand size. The measurement order began with two measurements of the left hand’s grip strength, recording the higher value. Then, two measurements of the right hand’s grip strength were taken, recording the higher value. The participants were instructed, “We will measure the grip strength twice on each side, left and right. Please hold the grip strength meter with your hand and place your elbow at the side of your body in contact.”

### 2.4. Data analysis

The word dictation task was analyzed in terms of linguistic, visuospatial, and kinematic aspects. In the linguistic aspect, a correct response was scored 1 point when all graphemes that make up the word were spelled accurately. If a linguistic error was made in any of the graphemes, 0 points were given. A total of 60 points were assigned, with 20 points each for regular words, irregular words, and non-words.

The types of linguistic errors were analyzed as follows:

(1) In all word conditions, the responses were analyzed by dividing substitution [e.g., 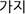 (/gaji/) →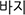 (/baji/)], addition [e.g., 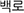 (/baengno/) →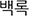 (/baengnok)], and omission [e.g., 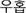 (/uhop/) 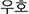 (/uho/)] errors based on the grapheme unit. Responses answered as “I don’t know” (DK) were considered as other errors. If the participant self-corrected after showing an erroneous response during the writing performance, it was specified as “correction” and the frequency was measured. In addition, the ratio (%) of the number of participants who showed correction in each group was qualitatively analyzed.
(2) In the irregular word condition, the patient’s responses were analyzed by classifying them into phonologically implausible errors (PIE) and phonologically plausible errors (PPE). PIE was defined as cases in which the participant’s spelling performance did not match the phonology of the target word or had an inappropriate form [e.g., 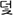 “(/deot/)→” 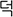 “(/deok/)”]. PPE was defined as a case in which the participant’s spelling performance was in the proper form with the phoneme of the target word [e.g., 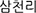 (/samcheonli/) →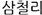 (/samcheolli/)].
(3) In the non-words condition, lexicalization errors were defined and analyzed when the target non-words were replaced with a meaningful word [e.g., 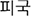 (/piguk/)→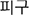 (pigu)].

Visuospatial aspects were analyzed based on the shape of letters or the combination of grapheme. Visuospatial errors refer to characters that cannot be seen in existing Korean alphabets and syllables, or awkward combinations. Therefore, all words were divided into “stroke level” and “grapheme level” and their frequency and type were analyzed. At the stroke level, it was divided into addition and omission errors. Stroke addition and stroke omission were regarded as visuospatial errors only when they were not replaced by another grapheme of Hangeul. At the grapheme level, grapheme combination errors were analyzed. A combination error of grapheme means an error in which each grapheme constituting one syllable maintains the form of a Korean grapheme, but the combination is incorrect and is combined in a form that does not conform to the rules of Hangeul syllable (Supplementary Table 2).

In terms of kinematics, pen pressure writing speed was checked. Pen pressure meant the pressure applied when writing a letter on a tablet notebook, and was measured as the average value of changing pen pressure from the point the pen touched the screen to the point the pen was removed to write a single stroke. The range of pen pressure was measured within the range of 0–4,096 levels, which is the range that can be measured on a tablet notebook, and the measured value was automatically converted into the range of 0.00001–1 levels in the software and used. The pen pressure of “word dictation” was measured as the average value of the pen pressure values measured for each stroke while writing the target word. Writing speed was measured by taking into account the length of each stroke (length) and the time taken to write it (duration), which are automatically measured by the software. The speed (mm/ms) was calculated by dividing the length of each stroke (measured in mm from the point the pen touched the tablet notebook screen to the point it was lifted) by the time taken to write it (measured in ms), for each stroke required to write a single character (Lee et al., 2020). The speed of “word dictation” was measured by the speed value obtained by adding the total time required while writing the target word and the total stroke length.

### 2.5. Statistical analysis

To find out the interaction (to see whether the performance aspects or patterns among groups differed depending on the word type), repeated one-way analysis of variance (ANOVA) was performed using the SPSS (version 26) program. ANOVA and Bonferroni *post-hoc* tests were performed to examine the differences among groups. A paired *t*-test or repeated one-way ANOVA was performed for within-group differences. To control for type I error across multiple testing, the significance probability was corrected with Bonferroni correction.

## 3. Results

### 3.1. Linguistic performance

#### 3.1.1. Number of correct responses

There was a significant difference between groups in the total number of correct responses in the word dictation task [*F*_(2,87)_ = 22.069, *p* < 0.001]. As a result of the *post-hoc* analysis, the number of correct responses was lower in the aMCI (*p* = 0.002) and AD (*p* < 0.001) groups than in the HC group, and lower in the AD (*p* = 0.005) group than in the aMCI group.

Evaluating each word type, in terms of the number of correct responses, the interaction effect according to group x word type [*F*_(3,134)_ = 3.153, *p* = 0.026], the main effect according to word type between groups [*F*_(2,87)_ = 22.069, *p* < 0.001], and the main effect according to word type within groups [*F*_(2,134)_ = 92.447, *p* < 0.001] were significant.

As a result of examining the performance of each group, the number of correct responses to regular words was lower in the AD (*p* < 0.001) group than in the HC group, but there was no significant difference between the HC group and the aMCI group. The AD (*p* < 0.001) group showed a lower number of correct responses than the aMCI group. The number of correct responses to irregular words was lower in the aMCI (*p* = 0.012) and AD (*p* < 0.001) groups than in the HC group, and the AD (*p* = 0.009) group showed lower numbers of correct responses than the aMCI group. The number of correct responses to non-words was lower in the aMCI (*p* = 0.004) and AD (*p* < 0.001) groups than in the HC group, and there was no significant difference between the aMCI and AD groups.

As a result of examining the performance within each group, the HC group performed lower on irregular words (*t* = 4.675, *p* < 0.001) and non-words (*t* = 6.017, *p* < 0.001) than on regular words, and lower on non-words (*t* = 3.337, *p* = 0.006) than on irregular words. The aMCI group had lower performance on irregular words (*t* = 6.412, *p* < 0.001) and non-words (*t* = 10.455, *p* < 0.001) than regular words, and lower performance on non-words (*t* = 3.708, *p* = 0.003) than irregular words. The AD group performed lower on irregular words (*t* = 6.319, *p* < 0.001) and non-words (*t* = 7.794, *p* < 0.001) than regular words, but there was no significant difference between irregular words and non-words (Table 2).

**TABLE 2:** Comparison of the number of correct responses in word dictation among the groups.

Word type	HC (*n* = 33)	aMCI (*n* = 31)	AD (*n* = 26)	Total (*n* = 90)	*F*	*Post-hoc*
Total word	51.82 (5.07)	45.45 (6.73)	39.15 (9.88)	45.97 (8.81)	20.069***	1 > 2, 1 > 3, 2 > 3
Regular word	18.88 (1.08)	18.13 (1.52)	16.08 (2.80)	17.81 (2.18)	17.085***	1 = 2, 1 > 3, 2 > 3
Irregular word	17.48 (1.95)	15.00 (3.24)	12.27 (4.69)	15.12 (3.94)	17.495***	1 > 2, 1 > 3, 2 > 3
Non-word	15.45 (3.49)	12.32 (3.66)	10.81 (4.23)	13.03 (4.21)	11.864***	1 > 2,1 > 3, 2 = 3

HC, healthy control; aMCI, amnestic mild cognitive impairment; AD, Alzheimer’s disease; 1 = HC; 2 = aMCI; 3 = AD; values are presented as mean (SD). ***p < 0.001.

#### 3.1.2. Number of errors by error type

In the word dictation task, in terms of the number of errors, the interaction effect according to group x error type [*F*_(2,95)_ = 20.014, *p* < 0.001], the main effect according to error type between groups [*F*_(2,87)_ = 20.729, *p* < 0.001], and the main effect according to error type within groups [*F*_(1,95)_ = 263.296, *p* < 0.001] were significant. As a result of examining the performance between each group, there were more grapheme substitution errors in the aMCI (*p* = 0.009) and AD (*p* < 0.001) groups than in the HC group, and more in the AD (*p* = 0.002) group than in the aMCI group. Grapheme addition errors were more in the aMCI (*p* = 0.021) and AD groups (*p* = 0.01) than in the HC group, and there was no significant difference between the aMCI and AD groups. Grapheme omission errors were more in the AD (*p* < 0.001) group than in the HC group, and more in the AD (*p* = 0.005) than in the aMCI group. There was no significant difference between the HC group and the aMCI group. There was no significant difference in DK error between the groups.

Performance evaluation within each group revealed that the HC group had more grapheme substitution errors than grapheme addition, grapheme omission, and DK errors [(grapheme addition *t* = 8.317, *p* < 0.001), (grapheme omission *t* = 8.700, *p* < 0.001), (DK *t* = 8.714, *p* < 0.001)]. There were more grapheme addition (*t* = 3.218, *p* = 0.018) errors than DK, and there was no significant difference between grapheme addition and grapheme omission errors, and grapheme omission and DK errors. The aMCI group showed more grapheme substitution errors than grapheme addition, grapheme omission, and DK errors [(grapheme addition *t* = 10.401, *p* < 0.001), (grapheme omission *t* = 10.948, *p* < 0.001), (DK *t* = 10.976, *p* < 0.001)]. There were more grapheme addition errors than grapheme omission and DK errors [(grapheme omission *t* = 3.020, *p* = 0.03), (DK *t* = 6.526, *p* < 0.001)]. There were more grapheme omission errors (*t* = 3.230, *p* = 0.018) than DK errors. The AD group had more grapheme substitution errors than grapheme addition, grapheme omission, and DK errors [(grapheme addition *t* = 9.115, *p* < 0.001), (grapheme omission *t* = 9.530, *p* < 0.001), (DK *t* = 9.245, *p* < 0.001)]. There were more grapheme addition errors (*t* = 4.660, *p* < 0.001) and grapheme omission errors (*t* = 3.931, *p* = 0.006) than DK errors. There was no significant difference between grapheme addition errors and grapheme omission errors (Table 3).

**TABLE 3:** Frequency of error types among the groups.

Error type	HC (*n* = 33)	aMCI (*n* = 31)	AD (*n* = 26)	Total (*n* = 90)	*F*	*Post-hoc*
Grapheme substitution	8.27 (5.46)	15.03 (7.65)	23.38 (12.91)	14.97 (10.70)	21.018***	1 > 2, 1 > 3, 2 > 3
Grapheme addition	0.76 (1.12)	1.74 (1.44)	1.88 (1.73)	1.42 (1.50)	5.739**	1 < 2, 1 < 3, 2 = 3
Grapheme deletion	0.36 (0.60)	0.74 (1.13)	2.08 (2.54)	0.99 (1.70)	9.376***	1 = 2, 1 < 3, 2 < 3
DK (do not know)	0.09 (0.29)	0.10 (0.30)	0.23 (0.71)	0.13 (0.46)	0.838	N/A

HC, healthy control; aMCI, amnestic mild cognitive impairment; AD, Alzheimer’s disease; 1 = HC; 2 = aMCI; 3 = AD; N/A = not applicable; values are presented as mean (SD). **p < 0.01, ***p < 0.001.

#### 3.1.3. PPE and PIE errors

For errors in the irregular word condition, the interaction effect according to group x error type [*F*_(2,87)_ = 7.229, *p* = 0.001], the main effect according to error type between groups [*F*_(2,87_*_)_* = 18.040, *p* < 0.001], and the main effect according to error type within groups [*F*_(1,87)_ = 40.334, *p* < 0.001] were significant. As a result of examining the performance of each group, PPE errors were higher in the aMCI (*p* = 0.001) and AD (*p* < 0.001) groups than in the HC group, and there was no significant difference between the aMCI and AD groups. PIE errors were more in the AD (*p* < 0.001) group than in the HC group, and more in the AD (*p* < 0.001) group than in the aMCI group. There was no significant difference between the HC group and the aMCI group.

As a result of examining performance within each group, the HC group showed more PIE errors (*t* = −5.392, *p* < 0.001) than PPE errors. In the aMCI group, there was no significant difference between PPE and PIE errors. The AD group showed more PIE errors (*t* = −4.066, *p* < 0.001) than PPE errors (Table 4).

**TABLE 4:** Frequency of PPE and PIE error types among the groups.

Error type	HC (*n* = 33)	aMCI (*n* = 31)	AD (*n* = 26)	Total (*n* = 90)	*F*	*Post-hoc*
PPE	0.58 (0.75)	2.03 (1.96)	2.31 (1.67)	1.58 (1.70)	11.465***	1 < 2, 1 < 3, 2 = 3
PIE	1.82 (1.47)	2.68 (1.78)	5.35 (3.89)	3.13 (2.87)	15.328***	1 = 2, 1 < 3, 2 < 3

HC, healthy control; aMCI, amnestic mild cognitive impairment; AD, Alzheimer’s disease; PPE, phonological plausible error; PIE, phonological implausible error; 1 = HC; 2 = aMCI; 3 = AD; values are presented as mean (SD). ***p < 0.001.

#### 3.1.4. Lexicalization error

In the non-words condition, lexicalization errors showed significant differences between the groups [*F*_(2,87)_ = 15.719, *p* < 0.001]. As a result of the *post-hoc* analysis, the number of lexicalization errors was higher in the aMCI (*p* < 0.001) and AD (*p* < 0.001) groups than in the HC group, but there was no significant difference between the aMCI and AD groups (Table 5).

**TABLE 5:** Frequency of lexicalization errors among the groups.

Error type	HC (*n* = 33)	aMCI (*n* = 31)	AD (*n* = 26)	Total (*n* = 90)	*F*	*Post-hoc*
Lexicalization	1.64 (1.54)	3.58 (1.79)	3.77 (1.66)	2.92 (1.92)	15.719***	1 < 2, 1 < 3, 2 = 3

HC, healthy control; aMCI, amnestic mild cognitive impairment; AD, Alzheimer’s disease; 1 = HC; 2 = aMCI; 3 = AD; values are presented as mean (SD). ***p < 0.001.

#### 3.1.5. Self-correction frequency

There was a significant difference between groups in the frequency of self-corrections observed in all word dictation tasks [*F*_(2,87)_ = 10.996, *p* < 0.001]. As a result of the *post-hoc* analysis, self-correction was higher in the aMCI group than in the HC (*p* < 0.001) and AD (*p* = 0.046) groups, but there was no significant difference between the HC and AD groups (Table 6).

**TABLE 6:** Frequency of self-correction errors among the groups.

	HC (*n* = 33)	aMCI (*n* = 31)	AD (*n* = 26)	Total (*n* = 90)	*F*	*Post-hoc*
Self-correction	0.42 (0.79)	2.35 (1.99)	1.27 (1.97)	1.33 (1.82)	10.996***	1 < 2, 1 = 3, 3 < 2

HC, healthy control; aMCI, amnestic mild cognitive impairment; AD, Alzheimer’s disease; 1 = HC; 2 = aMCI; 3 = AD; values are presented as mean (SD). ***p < 0.001.

### 3.2. Visuospatial performance

#### 3.2.1. Number of errors at the stroke level

In terms of stroke level errors in the word dictation task, the interaction effect according to group x stroke level was not significant, but the main effect according to the stroke level between groups [*F*_(2,87)_ = 12.196, *p* < 0.001] and within the group was significant [*F*_(1,87)_ = 6.233, *p* = 0.014]. Evaluating the performance of each group showed that stroke addition errors were higher in the AD group than in the HC (*p* < 0.001) and aMCI (*p* < 0.001) groups, and there was no significant difference between the HC and aMCI groups. Stroke omission errors were more frequent in the AD group (*p* = 0.017) than the HC group, and there was no significant difference between the HC group and the aMCI group, and between the aMCI group and the AD group. Examining the performance within each group showed that there was no significant difference between stroke addition and stroke omission errors in all groups (Table 7).

**TABLE 7:** Stroke-level error frequency among the groups.

Error type	HC (*n* = 33)	aMCI (*n* = 31)	AD (*n* = 26)	Total (*n* = 90)	*F*	*Post-hoc*
Stroke addition	0.18 (0.39)	0.29 (0.59)	1.46 (1.88)	0.59 (1.22)	11.752***	1 = 2, 1 < 3, 2 < 3
Stroke omission	0.09 (0.29)	0.19 (0.48)	0.73 (1.49)	0.31 (0.90)	4.444*	1 < 3, 1 = 2, 2 = 3

HC, healthy control; aMCI, amnestic mild cognitive impairment; AD, Alzheimer’s disease; 1 = HC; 2 = aMCI; 3 = AD; values are presented as mean (SD). *p < 0.05, ***p < 0.001.

#### 3.2.2. Number of errors at grapheme level

In the word dictation task, there was a significant difference between the groups in grapheme combination errors [*F*_(2,87)_ = 4.199, *p* = 0.018]. The *post-hoc* analysis revealed that the AD group (*p* = 0.014) had more grapheme combination errors than the HC group, but there was no significant difference between the HC group and the aMCI group, and between the aMCI group and the AD group (Table 8).

**TABLE 8:** Graphemic-level error frequency among the groups.

Error type	HC (*N* = 33)	aMCI (*N* = 31)	AD (*N* = 26)	Total (*N* = 90)	*F*	*Post-hoc*
Grapheme combination	0.12 (0.42)	0.61 (1.34)	1.23 (2.29)	0.61 (1.51)	4.199**	1 = 2, 1 < 3, 2 = 3

HC, healthy control; aMCI, amnestic mild cognitive impairment; AD, Alzheimer’s disease; 1 = HC; 2 = aMCI; 3 = AD; values are presented as mean (SD). **p < 0.01.

### 3.3. Kinematic performance

#### 3.3.1. Pen pressure

There was no significant difference in pen pressure between groups in a total of 60-word dictation tasks (Table 9).

**TABLE 9:** Pen pressure in the total 60-word dictation task among the groups.

	HC (*n* = 33)	aMCI (*n* = 31)	AD (*n* = 26)	Total (*n* = 90)	*F*
Total 60-word	0.54145 (0.14541)	0.50432 (0.17654)	0.51212 (0.17353)	0.52019 (0.16383)	0.449

HC, healthy control; aMCI, amnestic mild cognitive impairment; AD, Alzheimer’s disease; values are presented as mean (SD).

#### 3.3.2. Writing speed

There was a significant difference between groups in terms of the writing speed of a total of 60 word dictation tasks [*F*_(2,87)_ = 5.396, *p* = 0.006]. *Post-hoc* analysis showed that the aMCI group (*p* = 0.024) and the AD group (*p* = 0.015) were slower than the HC group, but there was no significant difference between the aMCI group and the AD group (Table 10).

**TABLE 10:** Writing speed in the total 60-word dictation task among the groups.

	HC (*n* = 33)	aMCI (*n* = 31)	AD (*n* = 26)	Total (*n* = 90)	*F*	*Post-hoc*
Total 60-word	0.07379 (0.03718)	0.05492 (0.02091)	0.05274 (0.01971)	0.06121 (0.02910)	5.396**	1 > 2, 1 > 3, 2 = 3

HC, healthy control; aMCI, amnestic mild cognitive impairment; AD, Alzheimer’s disease; 1 = HC; 2 = aMCI; 3 = AD; values are presented as mean (SD). **p < 0.01.

#### 3.3.3. Pen pressure according to syllable length

In terms of pen pressure according to syllable length in the word dictation task, the interaction effect according to group x syllable length and the main effect according to syllable length between groups were not significant, but the main effect according to syllable length within the group was significant [*F*_(2,156)_ = 19.909, *p* < 0.001]. The post-analysis showed that both the HC and AD groups had an increasing pattern of pressure with increasing syllable length, and the HC group showed a significant increase in pressure at 4 syllables (*t* = −3.963, *p* < 0.001) compared to 1 syllable. In the AD group, there was a significant increase in pressure at 2 and 4 syllables compared to 1 syllable (1 syllable and 2 syllables *t* = −3.140, *p* = 0.024, 1 syllable and 4 syllables *t* = −3.758, *p* = 0.006). There was no significant difference in all syllables in the aMCI group (Table 11).

**TABLE 11:** Pen pressure according to syllable length among the groups.

Syllable	HC (*n* = 33)	aMCI (*n* = 31)	AD (*n* = 26)	Total (*n* = 90)	*F*
1 syllable	0.52914 (0.14596)	0.48647 (0.17765)	0.47741 (0.17954)	0.49949 (0.16698)	0.436
2 syllables	0.54243 (0.14600)	0.50516 (0.18185)	0.51567 (0.17277)	0.52186 (0.16572)	0.656
3 syllables	0.54360 (0.14736)	0.51296 (0.17182)	0.51113 (0.17799)	0.52367 (0.16399)	0.685
4 syllables	0.55366 (0.14214)	0.50795 (0.16977)	0.52588 (0.17484)	0.52989 (0.16109)	0.525

HC, healthy control; aMCI, amnestic mild cognitive impairment; AD, Alzheimer’s disease; values are presented as mean (SD).

#### 3.3.4. Writing speed according to syllable length

In terms of writing speed according to syllable length of the word dictation task, the interaction effect according to group x syllable length was not significant, but the main effects according to syllable length between groups [*F*_(2,87)_ = 4.028, *p* = 0.021] and syllable length within groups [*F*_(2,159)_ = 19.422, *p* < 0.001] were significant. Evaluating the performance between each group revealed that the aMCI group exhibited slower writing speed than the HC group from 1 to 3 syllables, but there was no significant difference between the HC and AD groups, or between the aMCI and AD groups [(1 syllable *p* = 0.008), (2 syllables *p* = 0.026), (3 syllables *p* = 0.031)]. There was no significant difference among the groups at 4 syllables.

Evaluating performance within each group showed that the writing speed of the HC group increased significantly only in 3 syllables rather than 2 syllables (*t* = −3.217, *p* = 0.018). In the aMCI group, as the syllable length increased, the writing speed significantly increased [(1 syllable and 2 syllables *t* = −3.809, *p* = 0.006), (1 syllable and 3 syllables *t* = −5.442, *p* < 0.001), (1 syllable and 4 syllables *t* = −5.086, *p* < 0.001), (2 syllables and 3 syllables *t* = −4.677, *p* < 0.001), (2 syllable and 4 syllables *t* = −3.727, *p* = 0.006)]. In the AD group, there was no significant difference in all syllables (Table 12).

**TABLE 12:** Writing speed according to syllable length among the groups.

Syllable	HC (*n* = 33)	aMCI (*n* = 31)	AD (*n* = 26)	Total (*n* = 90)	*F*	*Post-hoc*
1 syllable	0.07123 (0.03472)	0.05017 (0.02083)	0.05588 (0.02278)	0.05954 (0.02848)	5.104**	1 > 2, 1 = 3, 2 = 3
2 syllables	0.07355 (0.03865)	0.05387 (0.02148)	0.05943 (0.02315)	0.06269 (0.03028)	3.819*	1 > 2, 1 = 3, 2 = 3
3 syllables	0.07604 (0.03814)	0.05661 (0.02274)	0.06114 (0.02417)	0.06504 (0.03058)	3.742*	1 > 2, 1 = 3, 2 = 3
4 syllables	0.07432 (0.03584)	0.05726 (0.02244)	0.06107 (0.02276)	0.06462 (0.02889)	3.214	N/A

HC, healthy control; aMCI, amnestic mild cognitive impairment; AD, Alzheimer’s disease; 1 = HC; 2 = aMCI; 3 = AD; N/A = not applicable; values are presented as mean (SD). *p < 0.05, **p < 0.01.

#### 3.3.5. Comparison of pen pressure and writing speed according to syllable length in a copying task

By measuring the pen pressure according to syllable length in the copying task, we found that there were no significant differences within the HC, aMCI, and AD groups (Supplementary Table 3). Measuring the writing speed according to syllable length in the copying task showed that there were no significant differences within the HC, aMCI, and AD groups (Supplementary Table 4).

## 4. Discussion

### 4.1. Overview

In this study, we identified linguistic, visuospatial, and kinematic aspects through a word dictation task that could examine the decline in cognitive-linguistic-motor function in MCI and AD with Aβ deposition with high sensitivity.

### 4.2. Principal findings

First, in a word dictation task, we observed that the AD group showed lower linguistic performance compared to the HC and aMCI groups in regular words. Regular words were defined as words with lexical properties and regularity aspects, where the sound and letters correspond to each other in terms of grapheme-phoneme correspondence. Unlike irregular words and non-words, both lexical and phonological routes can be accessed, making it a relatively easy word condition (Lee et al., 2020). Therefore, it can be interpreted that while the performance is maintained until the aMCI stage, a decline in performance is observed from the AD stage owing to the difficulties in accessing not only the lexical route but also the phonological route (Sung, 2010). Irregular words are words that carry meaning like regular words but have a grapheme-phoneme inconsistency where the sound and letters do not correspond. Therefore, to perform irregular words, one must rely solely on the lexical route rather than the phonological route (Penniello et al., 1995; Sung, 2010; Yeon et al., 2018). In irregular words, we observed a staircase-like decline in performance as cognitive abilities gradually decreased from the aMCI stage to the AD stage. Here, we added a control for individuals who show cognitive impairment and decreased abilities in daily life, as well as positivity for Aβ deposition. Therefore, the results of this study suggest that damage to the lexical route can be caused by the aMCI stage with Aβ deposition, and it can be interpreted that it becomes more difficult to utilize the lexical route as one enters the dementia stage (Roeltgen, 2003). Non-words do not have a familiar meaning like regular or irregular words, and they have the characteristic of matching graphemes to phonemes, so they require activation of the phonological route rather than access to the semantic system (Yeon et al., 2018). In non-words, we observed that performance was lower in the aMCI and AD groups compared to the HC group. Traditionally, the processing of the phonological route is associated with the temporo-parietal lobe, Wernicke’s area, left fronto-temporal, Broca’s area in the left hemisphere, and perisylvian region (Rapcsak et al., 2009). Therefore, individuals with Aβ deposition and cognitive decline starting from the aMCI group may have difficulty utilizing the phonological route, and in the early stages of AD, writing processing via the phonological route is maintained at the level of the aMCI group.

Second, when examining the error types in the word dictation task, substitution errors gradually increased as the severity of cognitive impairment progressed from aMCI to AD, reflecting the overall decline in cognitive function. Addition errors were significantly more frequent in both aMCI and AD groups with cognitive impairment compared to the HC group, while omission errors were significantly more frequent only in the AD group. Specifically, substitution errors accounted for the highest proportion in all groups, and these results may reflect the unique characteristics of Hangeul. As Hangeul has a form of group writing in which each grapheme is gathered and combined, and the location of the initial, middle, and final consonant within a syllable is fixed, it has more visuospatial characteristics than the English alphabet arranged in a row (Yoon et al., 2010, 2012). Therefore, even if one grapheme is missing from a syllable, it is easier to recognize that the shape of the syllable has changed compared to the alphabet. Specifically, the patients may have recognized the position of the letter in the target word, but may not have known exactly what the letter was, and thus attempted to replace it with a letter they knew in order to maintain the shape of the target word, leading to more substitution errors compared to other error types. In this context, omission errors, which fail to maintain the shape of the syllable, may have been observed significantly more often only in the AD group, which had the most impaired cognitive function among the three groups.

Third, in irregular words, PPE errors increased from the aMCI stage, while PIE errors significantly increased as AD progressed. These results suggest that in the aMCI group, individuals utilized residual phonological pathways to perform writing tasks, whereas in the AD stage, difficulties with phonological pathways were exacerbated, leading to errors unrelated to the phonology of the target word. In non-words, lexicalization errors increased from the aMCI stage. This may be interpreted as a pattern in which incomplete lexical pathways lead to the production of meaning words that remain within the internal lexicon during the processing of non-words.

Fourth, as a result of comparing the frequency of correction between groups during word dictation, the aMCI group showed significantly more self-correction than the HC and AD groups (Supplementary Table 5). Considering the proportion of participants who made corrections, the NC group had 9 out of 33 participants (27.28%), the AD group had 10 out of 26 participants (38.46%), while the aMCI group had 25 out of 31 participants (80.65%), indicating a more than two-fold higher rate than the other two groups. This can be explained in relation to the attitudes and meta cognition of the participants shown in the word dictation test. In contrast to the HC and AD groups, the aMCI group was observed to constantly confirm the accuracy of their responses with the examiner during the task. This implies that the aMCI group is capable of monitoring their performance and asking for feedback, which is one of the meta cognition abilities related to executive control. Self-monitoring, a cognitive strategy related to higher cognitive abilities, involves systematically observing and recording one’s own behavior (Shapiro and Cole, 1994). In writing, it can be seen as checking whether one understands the target word they are writing. Therefore, self-correction during word dictation could reflect the use of goal recognition and cognitive strategies to resolve errors (Shapiro and Cole, 1994).

Fifth, visuospatial errors occurred significantly more frequent in the AD group. Damage to neurons in the brain areas including the temporal-parietal areas, which are responsible for visuospatial organization functions, can result in damage to the pathways responsible for these functions in the AD group (Zillmer and Spiers, 2001). Especially, the dorsal pathway located in the posterior parietal areas is associated with determining where the target is located and integrating information on spatial perception such as spatial configuration between targets and self-motion caused by the movement of the body (McFie et al., 1950; Trick and Silverman, 1991; Gilmore et al., 1994; Johnson et al., 2002; Kavcic et al., 2011). When this pathway is damaged, individuals may not be able to accurately depict an object and may transform the central axis or draw the object inaccurately or at a different location with intersecting lines. Additionally, they may rotate lines or parts of the object while drawing (McFie et al., 1950). A study analyzing the handwriting of individuals with CI reported that there was a possibility of changes in letter shapes (Cilia et al., 2021a). Therefore, the AD group’s ability to perceive and analyze spatial information may be impaired, resulting in the addition or omission of strokes during character processing. An error in the combination of grapheme means that a grapheme that exists in Hangeul is used, but the combination between grapheme is wrong and is expressed in the form of a syllable that does not exist in Hangeul. Grapheme combination errors were the most frequent in words composed of diphthongs (e.g., regular word “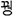,” non-words “
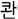
”). In terms of phonetics, vowels can be more subtle than vowels of other sounds in hearing and distinguishing sounds compared to consonants (e.g., “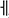,” “
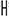
”). In particular, there were a lot of phoneme combination errors in singer vowels “
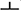
” and “
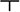
” (“
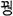
” single vowel “
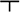
” was changed to “
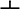
” and “
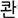
” single vowel “
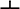
” was changed to “
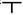
”). In a study (Byun, 2020) that confirmed the perceptual characteristics of /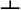/ and /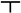/ in Korean with normal young adults in Korea, the difference between /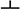/ and /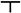/ vowels is not large in terms of oral opening and they are close, so it is difficult to distinguish them after hearing them. Therefore, a combination error of grapheme that was combined in a form that does not possibly conform to Korean rules was observed.

Sixth, as a result of comparing motor performance, the speed of the CI group (aMCI group and AD group) was slower than that of the HC group. Writing speed is influenced by various factors involved in performing movements, as well as cognitive processes involved in analyzing stimuli and organizing and planning responses during word dictation tasks (Van Galen, 1991; Jung, 2015). In tasks of writing a sentence dictation and repeated strokes (Delazer et al., 2021), mild AD patients exhibited slower writing speed and less automated movements compared to the HC group. They suggested that this could be caused by the deficits on not only central levels of processing but also the motor execution. In contrast, there was no significant difference in pressure between the groups, which is consistent with the previous study (Delazer et al., 2021). The pressure applied to the screen may be related to the basic strength of the hand. As a result, when grip strength was measured, no significant difference was observed between the groups, consistent with previous studies (Kluger et al., 1997). In the CI group, movement disorders may not be prominent in the early stages of the disease, and participants who participated in this study did not show major kinematic problems such as tremors or rigidity of body parts that could affect pen pressure. It can be interpreted that there was no difference between the groups.

Additionally, when comparing the results of motor performance according to syllable length (1 syllable, 2 syllables, 3 syllables, and 4 syllables), the motor performance according to syllable length differed according to the groups. In terms of pen pressure, a trend was observed in both the HC and AD groups that pen pressure increased as syllable length increased. The tablet screen used in this study has a lower coefficient of friction compared to paper, which can give the impression of slipping when writing (Gerth et al., 2016; Guilbert et al., 2019). Therefore, to compensate for the greater decrease in frictional force as the syllable length increases, the tension of the hand’s forearm muscles, shoulder, elbow, and muscles may have increased while performing writing tasks, leading to an increase in pen pressure (Bara and Gentaz, 2011; Kim et al., 2017). However, in the aMCI group, no significant difference in pen pressure was observed according to syllable length. This may be because frequent self-corrections during dictation tasks could cause interruptions in performance and may not reflect efforts to compensate for the decrease in frictional force. Therefore, it is possible that the absence of differences in pen pressure according to syllable length in the aMCI group, compared to the HC and AD groups, who wrote characters without frequent corrections, is due to the frequent corrections made by the aMCI group during writing tasks.

Notably, in terms of writing speed, the aMCI group was observed to have faster writing speed as syllable length increased. This can be attributed to the characteristics of the writing to dictation task. Unlike reading, in which visual cues are continuously provided, the dictation conducted in this study was a task in which the examiner listens to and writes down the contents. Accordingly, the dictation task, in which the audibly presented letters are temporarily stored and calculated by hand through the planning and programming stages of writing, required more working memory ability than reading (Kim et al., 2017). Since working memory is impaired in aMCI (Jang and Kim, 2014), the aMCI group may have compensated for their reduced working memory capacity by increasing the speed of hand movements to write longer syllables during the process of processing auditory presented words. However, in the case of the AD group, as the cognitive ability deteriorated further, the compensatory action for this did not occur properly; thus, it is assumed that the pattern of increasing the speed as the syllable lengthened was not observed. To confirm this assumption, we conducted an additional test in which the participants were asked to copy the same words as the ones used during dictation. Copying requires less working memory ability than dictation because the task allows the participants to continuously visualize the word while imitating it. Therefore, rather than confirming spelling knowledge, it can be said that it is a task that reflects more the visual compositional ability and motor ability required when performing writing tasks. Consequently, there were no changes in speed of the copying task even if the syllable length of words increased among all groups, including the aMCI group (Supplementary Table 4). The results of the copying task support the hypothesis that the demands on working memory in the spelling task may have affected writing speed with longer syllables.

### 4.3. Limitations

In future studies, it is necessary to analyze the neuroimaging and neuroanatomical correlations related to writing performance in the cognitively impaired group and healthy individuals. Moreover, the additional task was conducted among 20% of the total number of participants; thus, it is necessary to supplement the results through a full survey based on the total number of participants. While this study checked the grip strength as a basic motor ability of the hand related to pen pressure, it is necessary to also check basic motor abilities of the hand in relation to speed. Lastly, future improvement is needed as our data also reveal a gender bias, as reported that females have a higher prevalence of AD-type dementia compared to males (Gao et al., 1998; Kim and Han, 2012).

### 4.4. Clinical implications

In summary, the results of this study demonstrate that compared to those in the HC, patients in the aMCI group exhibited challenges in utilizing the lexical and phonological routes at an early stage, leading to a decline in performance with irregular words and non-words. As the disease progresses to the early stages of AD, the degree of impairment in phonological route utilization remained at the aMCI level, while the utilization of the lexical route became even more difficult. In terms of visuospatial aspects, patients with AD showed difficulties in adding or omitting strokes and combining graphemes. In terms of kinematic ability, both aMCI and AD groups showed a slower writing speed. This study is noteworthy and meaningful because it provided comprehensive results related to the writing performance of CI groups as “the First digital-based writing analysis study” that objectively and multidimensionally analyzed data collected using digitized software for patients with Aβ deposition. Our findings can be utilized as one of the cognitive-behavioral symptoms that can discriminate cognitive impairment caused by Aβ deposition. Furthermore, this study can serve as a clinical foundation for comparing and analyzing language, visuospatial, and motor abilities in other disease groups that show similar patterns of cognitive impairment as the CI group in the future.

## Data availability statement

The data underlying the results presented in this study contain potentially identifying or sensitive patient information and cannot be shared publicly due to restrictions imposed by the Institutional Review Board of Samsung Medical Center (SMC). However, the data are available from the Institutional Review Board of SMC (contact via https://www.e-irb.com:3443/index.jsp) for researchers who meet the criteria for access to confidential data.

## Ethics statement

The studies involving human participants were reviewed and approved by the Institutional Review Board of the Samsung Medical Center (No. 2021-12-068). The patients/participants provided their written informed consent to participate in this study.

## Author contributions

JY and DN: conceptualization. SA, HJ, HK, JY, and DN: methodology. SA: formal analysis and investigation and writing–original draft preparation. JY and DN: writing–review and editing. HJ, HK, and DN: resources. JY: supervision. All authors contributed to the article and approved the submitted version.
