# Supplementary material for: Linguistic, visuospatial, and kinematic writing characteristics in cognitively impaired patients with beta-amyloid deposition
Source: Front Aging Neurosci. 2023 Sep 11;15:1217746. doi: 10.3389/fnagi.2023.1217746 (PMC10518411; doi:10.3389/fnagi.2023.1217746)
Supplement: Supplementary file 1 [file Data_Sheet_1.docx]

Supplementary Material

Linguistic, Visuospatial, and Kinematic Writing Characteristics in Cognitively Impaired Patients with Beta-amyloid Deposition

Seo Kyung An, Hyemin Jang, Hee Jin Kim, Duk L. Na, and Ji Hye Yoon*

*** Correspondence:** Ji Hye Yoon: j.yoon@hallym.ac.kr

# Supplementary Tables

**Supplementary Table 1.** Stimuli of word dictation and copying tasks

| Stimuli of word dictation task | | | |
| --- | --- | --- | --- |
|  | Regular word | Irregular word | Non word |
| 1 | 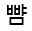(/ppyam/ means ‘cheek’) | 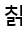(/chik/ means ‘[kudzu](https://en.dict.naver.com/#/entry/enko/8efabd69618c4536ae297f417d277cb5)’) | 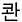(/kwan/) |
| 2 | 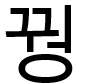 (/kkwong/ means ‘pheasant’) | 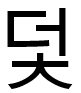 (/deot/ means ‘trap’) | 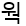(/wok/) |
| 3 | 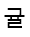(/gyul / means ‘mandarin’) | 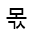(/mok/ means ‘share’) | 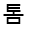(/tom/) |
| 4 | 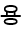(/yong/ means ‘dragon’) | 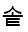(/sot/ means ‘Korean traditional caldron made of cast iron’) | 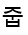(/jup/) |
| 5 | 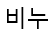(/binu/ means ‘soap’) | 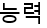(/neungnyeok/ means ‘ability’) | 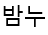(/bamnu/) |
| 6 | 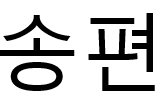(/songpyeon/ means ‘half-moon shape rice cake’) | 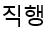(/jikaeng/ means ‘[direct](https://en.dict.naver.com/#/entry/enko/66da728f5e3348f8ac2d509acedc010e)’) | 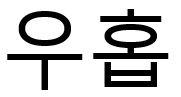(/uhop/) |
| 7 | 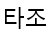(/tajo/ means ‘ostrich’) | 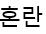(/hollan/ means ‘confusion’) | 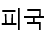(/piguk/) |
| 8 | 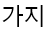(/gaji/ means ‘branch’) | 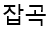(/japkkok/ means ‘[grain](https://en.dict.naver.com/#/entry/enko/a4cda5634b4d4fc6a5f94e76436d08e9)’) | 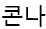(/konna/) |
| 9 | 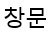(/changmun/ means ‘window’) | 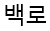(/baengno/ means ‘white heron’) | 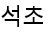(/seokcho/) |
| 10 | 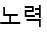(/noryeok/ means ‘effort’) | 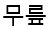(/mureup/ means ‘knee’) | 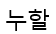(/nuhal/) |
| 11 | 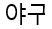(/yagu/ means ‘baseball’) | 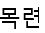(/mongnyeon/ means ‘magnolia’) | 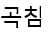(/gokchim/) |
| 12 | 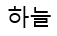(/haneul/ means ‘sky’) | 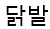(/dakppal/ means ‘chicken foot’) | 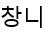(/changni/) |
| 13 | 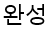(/wanseong/ means ‘completion’) | 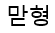(/matyeong/ means ‘the eldest brother’) | 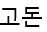(/godon/) |
| 14 | 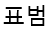(/pyobeom/ means ‘leopard’) | 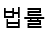(/beomnyul/ means ‘law’) | 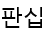(/pansip/) |
| 15 | 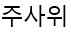(jusawi/ means ‘dice’) | 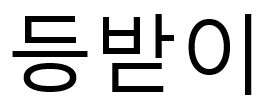(/deungbaji/ means ‘back rest’) | 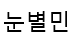(/nunbyeolmin/) |
| 16 | 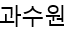(/gwasuwon/ means ‘orchard,’) | 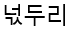(/neoktturi/ means ‘grumble’) | 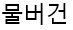(/mulbeogeon/) |
| 17 | 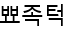(/ppyojokteok/ means ‘sharp chin’) | 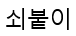(/soebuchi/ means ‘iron’) | 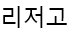(/rijeogo/) |
| 18 | 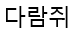(/daramjwi/ means ‘squirrel’) | 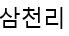(/samcheolli/ means ‘the whole land of Korea’) | 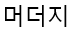(/meodeoji/) |
| 19 | 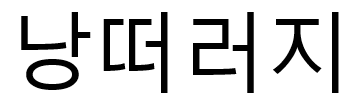 (/nangtteoleoji/ means ‘precipice’) | 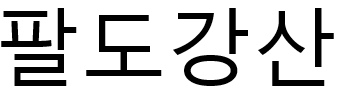(/palttogangsan/ means ‘the scenery of all parts of Korea’) | 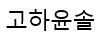(/gohayunsol/) |
| 20 | 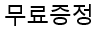(/muryojeungjeong/ means ‘giveaway gifts’) | 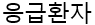(/eunggeupwanja/ means ‘emergency patient’) | 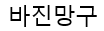(/bajinmanggu/) |
| Stimuli of copying task | | | |
|  | Regular word | Irregular word | Non word |
| 1 | 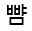(/ppyam/ means ‘cheek’) | 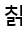(/chik/ means ‘[kudzu](https://en.dict.naver.com/#/entry/enko/8efabd69618c4536ae297f417d277cb5)’) | 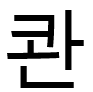(/kwan/) |
| 2 | 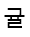(/gyul / means ‘mandarin’) | 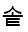(/sot/ means ‘Korean traditional caldron made of cast iron’) | 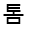(/tom/) |
| 3 | 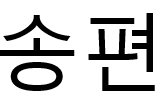 (/songpyeon/ means ‘half-moon shape rice cake’) | 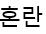(/hollan/ means ‘confusion’) | 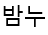(/bamnu/) |
| 4 | 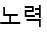(/noryeok/ means ‘effort’) | 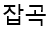(/japkkok/ means ‘[grain](https://en.dict.naver.com/#/entry/enko/a4cda5634b4d4fc6a5f94e76436d08e9)’) | 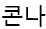(/konna/) |
| 5 | 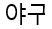(/yagu/ means ‘baseball’) | 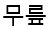(/mureup/ means ‘knee’) | 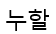(/nuhal/) |
| 6 | 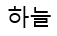(/haneul/ means ‘sky’) | 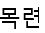(/mongnyeon/ means ‘magnolia’) | 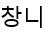(/changni/) |
| 7 | 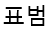(/pyobeom/ means ‘leopard’) | 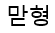(/matyeong/ means ‘the eldest brother’) | 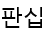(/pansip/) |
| 8 | 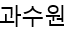(/gwasuwon/ means ‘orchard,’) | 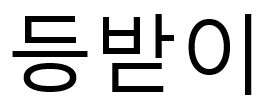(/deungbaji/ means ‘back rest’) | 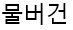(/mulbeogeon/) |
| 9 | 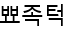(/ppyojokteok/ means ‘sharp chin’) | 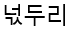(/neoktturi/ means ‘grumble’) | 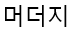(/meodeoji/) |
| 10 | 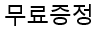(/muryojeungjeong/ means ‘giveaway gifts’) | 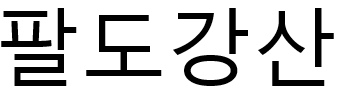(/palttogangsan/ means ‘the scenery of all parts of Korea’) | 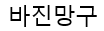(/bajinmanggu/) |

**Supplementary Table 2.** Examples of visuospatial errors at grapheme and stroke level

| Target word | Response |
| --- | --- |
| Examples of Stroke Omission Errors | |
| 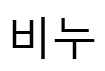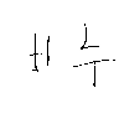 | |
| Examples of Stroke Addition Errors | |
| 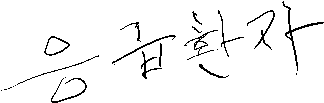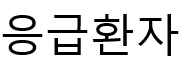 | |
| Examples of Grapheme Combination Errors | |
| 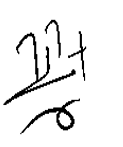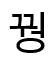 | |

**Supplementary Table 3**. Pen pressure of each group according to syllable length in copying

| Group | 1st syllable | | 2nd syllable | | 3rd syllable | | 4th syllable | |
| --- | --- | --- | --- | --- | --- | --- | --- | --- |
|  | Median | IQR | Median | IQR | Median | IQR | Median | IQR |
| HC (N=9) | 0.584 | 0.262 | 0.589 | 0.281 | 0.578 | 0.297 | 0.569 | 0.276 |
| aMCI (N=4) | 0.555 | 0.190 | 0.566 | 0.204 | 0.600 | 0.215 | 0.606 | 0.206 |
| AD (N=3) | 0.586 | 0.356 | 0.563 | 0.36 | 0.558 | 0.312 | 0.586 | 0.343 |

HC= healthy controls; aMCI = amnestic mild cognitive impairment; AD= Alzheimer’s disease; IQR= interquartile range.

**Supplementary Table 4**. Writing speed of each group according to syllable length in copying

| Group | 1st syllable | | 2nd syllable | | 3rd syllable | | 4th syllable | |
| --- | --- | --- | --- | --- | --- | --- | --- | --- |
|  | Median | IQR | Median | IQR | Median | IQR | Median | IQR |
| HC (N=9) | 0.238 | 0.211 | 0.235 | 0.177 | 0.260 | 0.183 | 0.288 | 0.184 |
| aMCI (N=4) | 0.198 | 0.121 | 0.192 | 0.124 | 0.208 | 0.101 | 0.205 | 0.113 |
| AD (N=3) | 0.170 | 0.287 | 0.196 | 0.269 | 0.191 | 0.307 | 0.202 | 0.287 |

HC= healthy controls; aMCI = amnestic mild cognitive impairment; AD= Alzheimer’s disease; IQR= interquartile range.

**Supplementary Table 5.** Example of self-correction in aMCI

| Target word | Response |
| --- | --- |
| 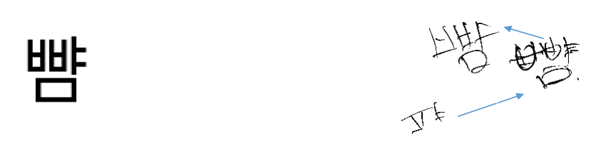 | |
| 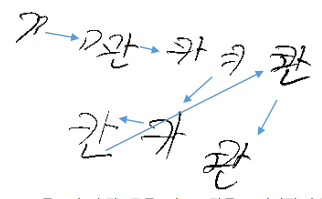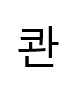 | |
| 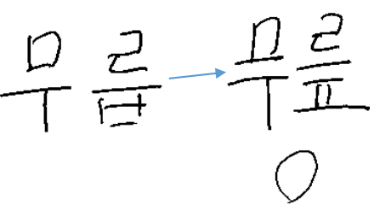 | |
|  | |
|  | |
